# The optimal dose of oxycodone in PCIA after laparoscopic surgery for gastrointestinal cancer in elderly patients: A randomized controlled trial

**DOI:** 10.3389/fsurg.2023.1111376

**Published:** 2023-03-17

**Authors:** Yanjun Zhou, Xinyi Huang, Huan Chang, Hongyu Sun, Wenxiu Xie, Ziye Pan, Fan Zhang, Qin Liao

**Affiliations:** ^1^ Department of Anesthesiology, The Third Xiangya Hospital, Central South University, Changsha, China; ^2^ Department of Anesthesiology, The People’s Hospital of Liuyang, Changsha, China

**Keywords:** gastrointestinal cancer, laparoscopic surgery, postoperative pain, oxycodone, patient controlled intravenous analgesia

## Abstract

**Objective:**

To explore the optimal bolus dose of oxycodone for patient controlled intravenous analgesia (PCIA) without background dose in elderly patients after laparoscopic surgery for gastrointestinal cancer.

**Methods:**

In this prospective, randomized, double-blind, parallel-controlled study, we recruited patients aged 65 years or older. They underwent laparoscopic resection for gastrointestinal cancer and received PCIA after surgery. Eligible patients were randomly divided into 0.01, 0.02, or 0.03 mg/kg group according to the bolus dose of oxycodone in PCIA. The primary outcome was VAS scores of pain on mobilization at 48 h after surgery. Secondary endpoints included the VAS scores of rest pain, the total and effective numbers of press in PCIA, cumulative dose of oxycodone used in PCIA, the incidence of nausea, vomiting and dizziness, as well as patients’ satisfaction at 48 h after surgery.

**Results:**

A total of 166 patients were recruited and randomly assigned to receive a bolus dose of 0.01 mg/kg (*n* = 55), 0.02 mg/kg (*n* = 56) or 0.03 mg/kg (*n* = 55) of oxycodone in PCIA. The VAS scores of pain on mobilization, the total and effective numbers of press in PCIA in 0.02 mg/kg group and 0.03 mg/kg group were lower than those in 0.01 mg/kg group (*P *< 0.05). Cumulative dose of oxycodone used in PCIA and patients’ satisfaction in 0.02 and 0.03 mg/kg groups were more than those in 0.01 mg/kg group (*P *< 0.01). The incidence of dizziness in 0.01 and 0.02 mg/kg groups was lower than that in 0.03 mg/kg group (*P *< 0.01). There were no significant differences in VAS scores of rest pain, the incidence of nausea and vomiting among three groups (*P *> 0.05).

**Conclusion:**

For elderly patients undergoing laparoscopic surgery for gastrointestinal cancer, 0.02 mg/kg bolus dose of oxycodone in PCIA without background infusion may be a better choice.

## Introduction

Gastrointestinal cancer is a common malignant tumor, and its prevalence and mortality increase with age, affecting millions of people around the world (1). Laparoscopic-assisted minimally invasive resection is the first-line treatment for the disease (2), offering the advantages of minimizing skin incision and reducing postoperative pain compared to open surgery (3). Nevertheless, postoperative pain, especially visceral pain, remains an important issue for patients undergoing laparoscopic surgery for gastrointestinal cancer (4).

PCIA based on μ-opioid receptor agonist, is widely used to relieve acute postoperative pain. Although PCIA has been applied, some patients still experienced visceral pain following abdominal surgery (5). Considering κ-opioid receptor has been found to be involved in visceral pain (6), it is possible that both μ and κ-opioid receptors agonist, such as oxycodone, may provide more effective analgesia than pure μ-opioid receptor agonist in patients following laparoscopic surgery for gastrointestinal cancer (4, 7). However, oxycodone in PCIA may cause serious side effects, including respiratory depression, apnea, bradycardia, hypotension, or even death (8). Especially elderly patients, their sensitivity to opioids is significantly increased. The demand for opioids in the elderly is lower, but the side effects are greater (9). Therefore, the dose adjustment of oxycodone in PCIA has become a challenge for elderly patients. Considering that oxycodone has an action time of more than 4 h (8), continuous background infusion may lead to overdose or obscure the actual needs of patients, especially in elderly patients (10, 11). We thought bolus dose and no background dose infusion of oxycodone in PCIA allowed elderly patients to achieve self-controlled analgesia, provided better titration and reduced side effects. Thus, we tried to explore the optimal bolus dose of oxycodone in PCIA without background infusion in elderly patients after laparoscopic surgery for gastrointestinal cancer.

To the best of our knowledge, there is little evidence regarding optimal dose of oxycodone in PCIA to elderly patients. In this prospective, randomized, double-blind study, we compared the analgesic efficacy, adverse events and patients’ satisfaction of oxycodone with different bolus doses in PCIA without background infusion among elderly patients who underwent laparoscopic surgery for gastrointestinal cancer.

## Methods

### Study design

This single-center, double-blinded, randomized control trial has been performed in the third Xiangya hospital of Central South University. The trial protocols were approved by the Ethics Committee of the third Xiangya hospital of Central South University (batch number: R16001) and registered at http://www.chictr.org.cn (ChiCTR-IPR-15006814, principal investigator: QL) before implementation. Written informed consent was obtained from all participants.

### Participants and recruitment

From July 2017 to September 2022, elderly patients who underwent elective laparoscopic surgery for gastrointestinal cancer in our hospital were assessed for eligibility on admission. Eligibility criteria were as follows: ≥65 years old; American Society of Anesthesiologists (ASA) physical status I–III; scheduled for elective laparoscopic surgery for gastrointestinal cancer and PCIA treatment. Written informed consent was obtained from each patient. Exclusion criteria were: (1) patients were reluctant to join the trial; (2) chronic pain before participating in the study; (3) long-term use of analgesics or other pain-related drugs, alcohol dependence; (4) patients with multiple comorbidities, including severe abnormal liver and kidney function, severe cardiovascular or cerebrovascular disease, psychiatric disorders; (5) allergy to oxycodone and its antagonist.

### Sample size

PASS software (version 15, NCSS, United States) was used for sample size calculation. We used the following settings to calculate it: *α* = 0.05, the test power 1-*β* = 0.8, two sided, and three groups allocation were equal. In the preliminary experiment, 60 elderly patients who underwent laparoscopic surgery for gastrointestinal cancer were enrolled. According to different bolus doses of oxycodone in PCIA, the above patients were randomly divided into 0.01, 0.02 or 0.03 mg/kg group, with 20 patients in each group. The VAS scores of pain on mobilization at 48 h after surgery in 0.01, 0.02 and 0.03 mg/kg group were 35.52 ± 13.02, 32.92 ± 11.05 and 28.65 ± 12.62, respectively. According to the results of the above preliminary experiment, we calculated that 50 patients were needed for each of three groups. Taking a 20% dropout rate into consideration, we needed to recruit 63 patients per group.

### Randomization and blinding

The enrolled patients were randomized to three groups by a biostatistician not involved in this trial using a computer program in a ratio of 1:1:1. Patients’ numbers were entered into SPSS 20.0 software (SPSS Inc., Chicago, United States) to generate a randomization scheme. Eligible patients were randomly assigned to 0.01, 0.02 or 0.03 mg/kg group. They received 0.01, 0.02, or 0.03 mg/kg bolus doses of oxycodone in PCIA in the absence of background infusion, respectively. Different doses of oxycodone in PCIA were prepared by a nurse who did not participate in the study. Neither researchers nor patients knew the group assignment.

### Anesthesia and PCIA protocol

The standardized and uniform anesthesia protocol was adopted in all groups. Premedication was not given to each patient. After patients were transported to the operating room, they were monitored pulse oximetry, electrocardiogram, heart rate, arterial blood pressure and arterial blood gas analysis. All patients’ anesthesia induction was performed with 0.04 mg/kg midazolam, 0.15 mg/kg cisatracurium besilate, 0.2 mg/kg etomidate, and 5 μg/kg fentanyl. After successful tracheal intubation, mechanical ventilation was performed. End-tidal PaCO_2_ was monitored and maintained between 35 and 45 mmHg. Fraction of inspired oxygen (FiO_2_) was set to 0.5. Anesthesia was maintained with a combination of intravenous and inhaled anesthesia according to the bispectral index (Bis EEG VISTA Covidien, America) and hemodynamics: inhalation of 1%–2% sevoflurane, intravenous infusion of propofol (4–6 mg/kg/h), remifentanil (5–15 μg/kg/h) and cisatracurium besilate (0.1–0.15 mg/kg/h) through micropumps. An intravenous bolus of 3 μg/kg fentanyl was used before skin incision. The anesthesiologist then decided to give fentanyl (1 μg/kg each time) according to the vital signs, but the maximum dose of any patients should not exceed 12 μg/kg to prevent the residual effects of fentanyl after surgery. 8 mg of ondansetron hydrochloride was administered intravenously to prevent postoperative nausea and vomiting before skin closure. 0.5% ropivacaine was used for local infiltration anesthesia in the wound after skin suture for wound analgesia. All patients’ surgeries were performed by two identical surgeons.

After surgery, patients were sent to the post-anesthesia care unit (PACU) for recovery. When the patient recovered breathing, neostigmine was used to antagonize muscle relaxation. The trachea was extubated when the patients were awake. Then we evaluated and recorded pain severity by VAS. If the patients felt moderate or severe pain (VAS 40–100), they were given 20 µg of fentanyl intravenously. Reassessed their pain 5 min later. If they still felt moderate or severe pain, intravenous bolus of 20 µg fentanyl was continued until their VAS was ≤30. PCIA was started when they perceived slight pain (VAS 10–30). We ensured that PCIA commenced at the same and mild pain level. The electronic-controlled analgesic pump (Renxian Medical, Jiangsu Province, China) contained 40 mg oxycodone in 0.9% normal saline for a total volume of 160 ml, and was programmed to no background infusion, PCIA dose of 0.01, 0.02 or 0.03 mg/kg bolus of oxycodone solution, with a lockout period of 5 min. All patients were told how to use the analgesic pump. PCIA was continued for 48 h after surgery. No other rescue drugs, except oxycodone, were used during the 48 h postoperatively.

### Outcome measurements

Demographic and clinical data, including patients’ age, gender, height, weight, ASA physical status, cancer’s type and stage, preoperative anxiety and depression scores, duration of surgery, and medical history were collected before surgery.

The primary outcome was the VAS scores of pain on mobilization at 48 h after surgery. The secondary outcomes included the VAS scores of rest pain, the total and effective numbers of press in PCIA, cumulative dose of oxycodone used in PCIA, adverse events (nausea, vomiting, and dizziness) and patients’ satisfaction within 48 h after surgery.

### Statistical analysis

SPSS 20.0 statistical software (SPSS Inc., Chicago, IL, United States) was used for statistical analysis. The Kolmogorov-Smirnov test was used to evaluate if the continuous variables followed a normal distribution. Normally distributed continuous variables were expressed as mean ± standard deviation (SD). Barlett’s test was employed to determine the variance homogeneity. One-way analysis of variance (ANOVA) was used to compare the three groups of continuous variables with normal distribution and homogeneous variance. If the differences were significant, the Student-Newman-Keuls *q* test was further used to compare the differences between pairwise groups. Numbers and/or percentages were used to describe the count data. The *χ*^2^ test was used to evaluate whether the count data exhibited significant differences among the three groups. A *P* value < 0.05 was considered statistically significant. Multiple comparisons of the count data (0.01 mg/kg group to 0.02 mg/kg group, 0.01 mg/kg group to 0.03 mg/kg group, 0.02 mg/kg group to 0.03 mg/kg group) were done for significant results, and the *α* lever was set at 0.017, following Bonferroni adjustment.

## Results

### Demographic data and clinical characteristics

**Figure 1:**
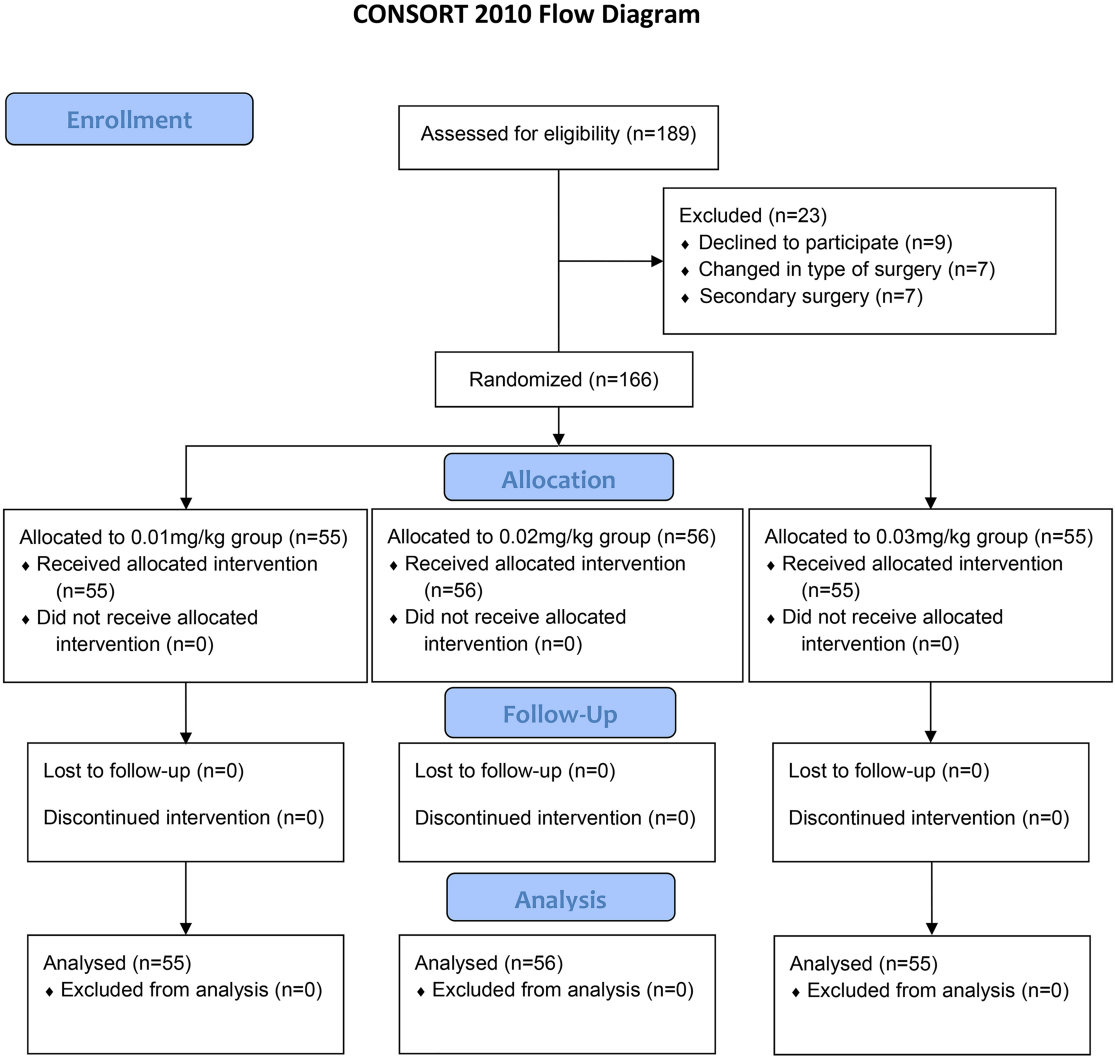
Consolidated standards of reporting trials (CONSORT) flow diagram of patients’ distribution.

A total of 189 patients who underwent laparoscopic surgery for gastrointestinal cancer were evaluated for eligibility before enrollment. Of these patients, nine patients declined to participate. Seven patients’ surgery type was converted to open surgery. Seven patients received a secondary surgery. Finally, 166 patients were enrolled in this study. After informed consent was obtained, eligible patients were prospectively randomized into 0.01 mg/kg group (*n* = 55), 0.02 mg/kg group (*n* = 56), or 0.03 mg/kg group (*n* = 55) (Figure 1). Their demographic and clinical characteristics were shown in Table 1. There were no significant differences in age, gender, height, weight, ASA physical status, stage of cancer, type of cancer, preoperative anxiety and depression, and duration of surgery among three groups (all *P *> 0.05).

**Table 1:** Demographic and clinical data of the patients.

	0.01 mg/kg group	0.02 mg/kg group	0.03 mg/kg group	*P* value
Age, years	72.55 ± 5.91	72.41 ± 4.57	70.78 ± 4.02	0.11
Gender, male/female	31/24	33/23	39/16	0.24
Height, cm	160.10 ± 6.97	161.10 ± 7.73	163.20 ± 8.23	0.10
Weight, kg	56.70 ± 10.07	57.72 ± 9.52	59.23 ± 9.91	0.40
ASA physical status I/II/III, *n*	3/15/37	5/23/28	4/21/30	0.45
Stage of cancer I/II/III, *n*	4/41/10	5/37/14	4/43/8	0.67
Type of cancer (stomach/colon/rectal), *n*	7/24/24	10/25/21	9/26/20	0.91
Preoperative anxiety scores	24.45 ± 4.37	25.39 ± 5.39	24.51 ± 4.85	0.53
Preoperative depression scores	25.55 ± 5.88	26.68 ± 7.07	24.87 ± 4.12	0.26
Duration of surgery, hours	3.85 ± 1.26	4.09 ± 1.48	4.06 ± 1.47	0.63

Data are presented as the mean ± standard deviation (SD) or count (percentage). ASA, American Society of Anesthesiologists.

### Analgesic effect and usage amount of oxycodone in PCIA

The VAS scores of pain on mobilization were significantly different among three groups at 48 h after surgery (*P *< 0.01). 0.02 mg/kg group and 0.03 mg/kg group had a lower scores of pain on mobilization than 0.01 mg/kg group (*P *< 0.05, respectively), but there was no significant difference between 0.02 mg/kg group and 0.03 mg/kg group (*P *> 0.05). There was no significant difference in VAS scores of rest pain at 48 h after surgery among three groups (*P *= 0.27).

During the first 48 h postoperatively, the total and effective numbers of press in PCIA among three groups were significantly different (*P *< 0.01, respectively). The total and effective numbers of press in 0.02 mg/kg group and 0.03 mg/kg group were both less than those in 0.01 mg/kg group (*P *< 0.05, respectively). There was no significant difference in total and effective numbers of press between 0.02 mg/kg group and 0.03 mg/kg group (*P *> 0.05).

Cumulative dose of oxycodone used in PCIA was significantly different among three groups within 48 h after surgery (*P *< 0.01). The dosage of oxycodone used in 0.02 mg/kg group and 0.03 mg/kg groups was significantly more than that in 0.01 mg/kg group (*P *< 0.05, respectively). There was no significant difference in dosage of oxycodone between 0.02 mg/kg group and 0.03 mg/kg group (*P *> 0.05) (Table 2).

**Table 2:** Primary outcome and secondary outcomes among three groups.

	0.01 mg/kg group (*n* = 55)	0.02 mg/kg group (*n* = 56)	0.03 mg/kg group (*n* = 55)	*P* value
**Primary outcome**
Scores of pain on mobilization	36.00 ± 13.14	30.30 ± 10.15	28.73 ± 12.77	<0.01
**Secondary outcomes**
Resting pain scores	13.27 ± 12.92	16.25 ± 13.01	12.73 ± 10.79	0.27
Total numbers of press, *n*	32.91 ± 24.00	20.16 ± 14.10	15.95 ± 10.66	<0.01
Effective numbers of press, *n*	23.11 ± 15.89	16.68 ± 10.53	13.44 ± 8.29	<0.01
Amount of oxycodone used in PCIA(mg)	12.72 ± 8.38	19.16 ± 11.44	22.07 ± 12.47	<0.01
Postoperative nausea, [*n*(%)]	8 (14.5%)	8 (14.3%)	10 (18.2%)	0.82
Postoperative vomiting, [*n*(%)]	3 (5.5%)	1 (1.8%)	3 (5.5%)	0.54
Postoperative dizziness, [*n*(%)]	2 (3.6%)	1 (1.8%)	13 (23.6%)	<0.01
Patients’ satisfaction				<0.01
Very satisfied, *n*(%)	12 (21.8%)	18 (32.1%)	19 (34.5%)	
Satisfied, *n*(%)	25 (45.5%)	35 (62.5%)	33 (60.0%)	
Neutral, *n*(%)	12 (21.8%)	3 (5.4%)	3 (5.5%)	
Dissatisfied, *n*(%)	6 (10.9%)	0 (0%)	0 (0%)	
Very dissatisfied, *n*(%)	0 (0%)	0 (0%)	0 (0%)	

Data are presented as mean ± standard deviation (SD) or count (percentage). PCIA, patient controlled intravenous analgesia.

### Adverse events

There was no significant difference in the incidence of nausea and vomiting among three groups during the first 48 h after surgery (*P *> 0.05, respectively). The incidence of dizziness in 0.01 mg/kg group and 0.02 mg/kg group was lower than that in 0.03 mg/kg group (*P *< 0.01, respectively). None of the patients had respiratory depression (Table 2).

### Patients’ satisfaction

In addition, there was significant difference in patients’ satisfaction within 48 h postoperatively among three groups (*P *< 0.01). Satisfaction of patients in 0.02 mg/kg group and 0.03 mg/kg group was significantly higher than that in 0.01 mg/kg group (*P *< 0.01, respectively). There was no significant difference in patients’ satisfaction between 0.02 mg/kg group and 0.03 mg/kg group (*P *> 0.05) (Table 2).

## Discussion

We conducted this prospective, randomized controlled study to compare the analgesic and adverse effects of varying doses of oxycodone in PCIA following laparoscopic surgery for gastrointestinal cancer in elderly patients. According to the results of this study, 0.02 mg/kg group and 0.03 mg/kg groups showed better analgesic effect and patients’ satisfaction than 0.01 mg/kg group. In addition, the incidence of dizziness in 0.02 mg/kg group was lower than that in 0.03 mg/kg group during the first 48 h after surgery. Thus, 0.02 mg/kg bolus dose of oxycodone in PCIA would be a better choice after laparoscopic surgery for gastrointestinal cancer in elderly patients.

Visceral pain is a large contributor of postoperative pain in patients with laparoscopic surgery for gastrointestinal cancer (4, 12, 13). κ-opioid receptor plays an important role in the mediation of visceral pain (6). Oxycodone, a dual agonist of μ and κ receptors (14), has been shown to provide effective analgesia for acute postoperative pain (15, 16), especially visceral pain. However, the safe and effective dose of oxycodone in PCIA following laparoscopic surgery for gastrointestinal cancer in elderly patients has not been determined. As we know, oxycodone is a long-acting analgesic, no background dose infusion in PCIA can reduce side effects in elderly patients. Therefore, we compared three different bolus doses of oxycodone, including 0.01, 0.02 and 0.03 mg/kg, expecting to find an optimal dose in PCIA without background infusion after laparoscopic surgery for gastrointestinal cancer in elderly patients.

VAS, the numbers of press and the satisfaction rate were used to assess the analgesic effect. VAS is a commonly used tool of pain intensity during the assessment of postoperative pain, including the pain at rest and on mobilization (17, 18). In our study, there was no significant difference in VAS scores of rest pain, but the VAS scores of pain on mobilization were significantly different among three groups at 48 h after surgery. 0.02 and 0.03 mg/kg groups had a lower pain scores on mobilization than 0.01 mg/kg group, which indicated that the pain on mobilization was not well controlled in 0.01 mg/kg group. The total and effective numbers of press in PCIA were significantly different during the first 48 h postoperatively among three groups. The total number and effective number of press in 0.02 mg/kg group and 0.03 mg/kg group were both significantly less than those in 0.01 mg/kg group. In 0.01 mg/kg group, the total number of press was about 33 times within 48 h after operation, which further indicated that the badly analgesic effect in 0.01 mg/kg group. And we also found that there was no significant difference of the VAS scores of pain on mobilization at 48 h after surgery between 0.02 mg/kg group and 0.03 mg/kg group, that is, 0.02 mg/kg of oxycodone could provide the same analgesic effect as 0.03 mg/kg of oxycodone.

Previous studies have shown that the adverse effects of oxycodone included nausea, vomiting, and dizziness (19–21). There was no significant difference in the incidence of postoperative nausea and vomiting among three groups in our study. However, the incidence of dizziness in 0.01 mg/kg group and 0.02 mg/kg group was lower than that in 0.03 mg/kg group during the first 48 h after surgery, which may be related to less dosage of oxycodone in 0.01 mg/kg group and 0.02 mg/kg group.

In addition, the satisfaction rate of patients in 0.02 mg/kg group and 0.03 mg/kg group was significantly higher than that in 0.01 mg/kg group. Although 0.01 mg/kg group had the lowest incidence of dizziness, it has a poor analgesic effect and a high number of press. The press number in 0.01 mg/kg group was close to once every 1.5 h, seriously affected the rest of patients and reduced the satisfaction rate of patients. Although 0.3 mg/kg group suffered highest incidence of dizziness, the degree of dizziness was slight and had little effect on the activity of the patients, which only occured when the patients got up. After the patients rested in bed for several minutes, dizziness relieved spontaneously. These patients in the 0.03 mg/kg group were satisfied with the good analgesic effect. There was no significant difference in patients’ satisfaction rate between 0.02 mg/kg group and 0.03 mg/kg group.

There are some limitations of this study. First, this was a single-center investigation. Second, we didn’t perform a multidimensional pain assessment in this study. Finally, whether postoperative analgesia with oxycodone without background infusion is suitable for all gastrointestinal surgical procedures (e.g., non-laparoscopic gastrointestinal procedures) needs to be further studied.

In summary, 0.02 mg/kg of oxycodone could provide the same analgesic effect and satisfaction as 0.03 mg/kg of oxycodone, but the incidence of dizziness was lower than that in the 0.03 mg/kg group. Therefore, 0.02 mg/kg bolus dose of oxycodone in PCIA without background infusion may be a better choice after laparoscopic surgery for gastrointestinal cancer in elderly patients.

## Data Availability

The raw data supporting the conclusions of this article will be made available by the authors, without undue reservation.
